# Pollen flow and paternity in an isolated and non-isolated black walnut (*Juglans nigra* L.) timber seed orchard

**DOI:** 10.1371/journal.pone.0207861

**Published:** 2018-12-04

**Authors:** Aziz Ebrahimi, Shaneka S. Lawson, Graham S. Frank, Mark V. Coggeshall, Keith E. Woeste, James R. McKenna

**Affiliations:** 1 Department of Forestry and Natural Resources, Purdue University, West Lafayette, IN, United States of America; 2 USDA Forest Service, Northern Research Station Hardwood Tree Improvement and Regeneration Center, West Lafayette, IN, United States of America; Austrian Federal Research Centre for Forests BFW, AUSTRIA

## Abstract

Artificial pollination of black walnut (*Juglans nigra* L.) is not practical and timber breeders have historically utilized only open-pollinated half-sib families. An alternate approach called “breeding without breeding,” consists of genotyping open-pollinated progeny using DNA markers to identify paternal parents and then constructing full-sib families. In 2014, we used 12 SSR markers to genotype 884 open-pollinated half-sib progeny harvested from two clonal orchards containing 206 trees, comprised of 52 elite timber selections. Seed was harvested in 2011 from each of two ramets of 23 clones, one upwind and one downwind, based on prevailing wind direction from the west—southwest. One orchard was isolated from wild black walnut and composed of forward selections while the other orchard was adjacent to a natural forest containing mature black walnut composed of backward selections. Isolation significantly increased within-orchard pollination (85%) of the progeny from the isolated orchard compared to 42% from the non-isolated orchard. Neither prevailing wind direction nor seed tree position in the orchard affected paternity patterns or wild pollen contamination. Genetic diversity indices revealed that progeny from both orchards were in Hardy–Weinberg equilibrium with very little inbreeding and no selfing. A significant level of inbreeding was present among the forward selected parents, but not the first generation (backward selected) parents. Some orchard clones failed to sire any progeny while other clones pollinated upwards of 20% of progeny.

## Introduction

Black walnut, *Juglans nigra* L., is a valuable timber tree native to eastern North America that also provides mast and habitat for wildlife and watershed protection [1]. Genetic improvement of black walnut began in the 1960s to preserve and propagate high-quality timber trees that were believed to have suffered from genetic erosion through excessive harvesting during the early twentieth century [2, 3].

Walnuts are monecious and wind pollinated, and although self-fertile, they predominately outcross through a reproductive system unique to *Juglans* species called heterodichogamy, in which pollen shed can occur either before (protandrous) or after (protogynous) pistillate flowers become receptive [4, 5]. Attempts to produce pedigreed progeny of black walnut (*Juglans nigra* L.) through controlled pollination were initiated several times over the last half-century, but all were largely unsuccessful [2, 4]. Efforts to isolate and bag pre-receptive female pistillate flowers often damage the flowers that develop on new shoots. Besides mechanical damage to the pistillate flowers, the viability of stored walnut pollen and its application requires careful control [6]. Finally, new walnut growth is very susceptible to spring frost damage, and pollination bags accentuate frost damage, killing both pistillate flowers and newly emerging shoots. [2] Reported that controlled pollination of 5,000 black walnut flowers, over 8 years of crossing, resulted in just 50 seedlings.

Successful breeding and deployment of improved black walnut requires an understanding and management of the dynamic factors associated with pollen dispersal patterns, phenology, genetic diversity, pollen contamination from wild trees, and inbreeding. [5] Reported patterns of pollen movement and paternal inheritance based on pollen source distance to optimize the placement of Persian walnut (*J*. *regia*) clones used as pollinizers in California orchards. Similar information is essential for proper management of black walnut timber seed orchards. Pollen of some tree species can maintain viability while traveling long distances [7], but effective pollinizer distances for Persian walnut orchards in California rarely exceed 457 meters [5].

Although mass selection or selection among open pollinated half-sib families are the predominant methods used in the early stages of forest tree breeding programs [8, 9, 10], genetic gains can be theoretically doubled by utilizing full-sib families [11]. The analysis of phenotypes of full-sib families permits estimation of the *specific* combining ability of various males to the same female. [12] demonstrated the effectiveness of “breeding without breeding” by comparing traditional cross pollination with genotyping open-pollinated progeny and assigning them to full-sib families. Experience has shown, however, that genetic gain from full-sib families does not necessarily double the gain from open-pollination, and less intensive open-pollinated breeding methods can rival the gains from controlled-pollination of forest trees [13]. Forest tree breeding of pines has shown the advantage of utilizing poly-mixed pollen sources composed of typically 10 male parents [14]. For walnut and other trees that are difficult to artificially pollinate, seed orchards can be arranged with consideration of phenology and genetic diversity to mimic such a polymix approach.

Open pollination breeding, besides its practical and economic advantages, can also provide reliable estimates of parental breeding values and broad sense heritability or *general* combining ability [11]. However, open pollination has several disadvantages, including the risk of pollen from unknown and undesired (wild) trees infiltrating the breeding population. Pollen contamination from wild trees or nearby conspecifics can reduce genetic gain nearly 50% in pines [15]. Furthermore, open pollination can lead to inbreeding, in the absence of methods to identify and control the male parents among selections [16]. Depending on specific breeding objectives, increased levels of inbreeding can be an important genetic management consideration.

Microsatellite marker-based techniques have been successfully used to estimate pollen flow by comparing segregation of genetic markers in both parents and offspring [17]. Exclusion probability for assigning paternity using these techniques is high if markers exhibit codominant inheritance and high levels of polymorphism [17, 18, 19]. In addition, these DNA tools allow the estimation of *effective* pollination since all progeny are fertilized, have germinated, and have survived in the field [18, 20, 21].

We genotyped all parents and a sample of progeny from two elite timber seed orchards using twelve polymorphic nuclear microsatellite loci with the objectives: 1) to examine genetic diversity and inbreeding of each orchard and its progeny; 2) to determine how isolation affects within-orchard pollination and; 3) to examine pollination dynamics based on distance and prevailing wind direction.

## Materials and methods

### Plant material and site information

#### Seed orchards

Two clonal black walnut seed orchards were propagated by grafting onto seedling rootstocks and planted in a completely randomized block design at the Lugar orchard (LO) (N°40.4263590, W°86.9613061) in West Lafayette, IN, established in 2002, and at the Vallonia Orchard (VO) (N°38.847, W°86.098) in Vallonia, IN, established in 1991. All materials from both orchards belong to the Hardwood Tree Improvement and Regeneration Center at Purdue University. Permission for use is required and must be confirmed by Purdue University, USA.

Clones were randomly assigned to locations in the orchards with 4–8 ramets per clone arranged in 4–8 blocks. Orchards were planted in 7 x 7 m (LO) and 6 x 6 m (VO) square plots. The VO orchard contains from 1 to 8 ramets of 37 forward selected clones and LO has 1 to 4 ramets of 15 first generation backward selected clones from the original Purdue University black walnut breeding program [2]. Diameter and height of all adult trees on each site were measured in 2017. The average height for trees in LO and VO orchards were 12.6 and 23 meters respectively. The average diameter for LO and VO trees were 22 and 34 cm.

#### Isolation

**Fig 1:**
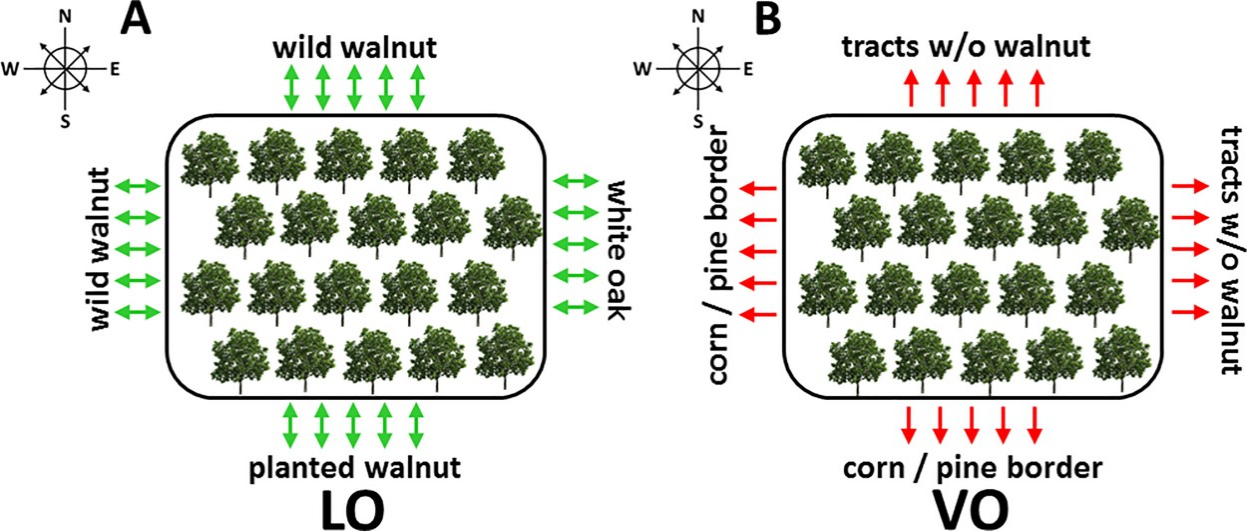
Schematic diagram of **(A)** Lugar (LO) and **(B)** Vallonia (VO) orchards. w/o = without.

The VO site was bordered to the south and west with white pine (*Pinus strobus*) and grain farms (**Fig 1**). Native forests occur to the east and north of VO and all wild walnuts within 2 km of the orchard were removed. The LO site was not isolated and is adjacent to a native forest with wild walnuts on the west and north sides, white oak to the east, and planted walnut to the south (500 m to 1km away) (**Fig 1**).

#### Seed

Nearly every tree at both orchard sites set seed in 2011 following a cool winter that provided ample chilling hours. A cold spring led to delayed bloom times. As temperatures rose above 20°C, blooming proceeded rapidly. Over 4,000 total seeds were harvested from all orchard trees. A limited sample of progeny from just two ramets per clone were sampled; one deemed “upwind”and one “downwind” based on the prevailing wind direction (**Table 1**). Each orchard tree was harvested individually by first clearing all early dropped seed and then shaking each tree with a mechanical tree shaker. Exceptional care was exercised to avoid mixing seed. Each seed lot was fall sown at the Indiana Department of Natural Resources, Division of Forestry Nursery, in Vallonia, Indiana, USA.

**Table 1:** Number of seedling progeny per orchard clone and prevailing wind direction.

Orchard	Maternal parent*	N	Number of within orchard male parents	Number of wild male parents	Prevailing wind direction
**Lugar Orchard**	55	23	4	19	upwind
**(LO)**	55	19	8	11	downwind
	119	19	7	12	upwind
	119	15	10	5	downwind
	130	14	4	10	upwind
	130	19	8	11	downwind
	132	11	6	5	upwind
	132	19	4	15	downwind
	158	19	8	11	upwind
	158	18	4	14	downwind
	178	24	15	9	upwind
	178	21	8	13	downwind
	205	19	10	9	upwind
	205	19	15	5	downwind
	288	13	7	5	upwind
	288	19	6	13	downwind
	297	22	7	15	upwind
	297	23	10	13	downwind
	316	24	12	12	upwind
	316	11	2	9	downwind
	644	4	4	0	upwind
	644	5	3	2	downwind
**Vallonia Orchard**	222	7	11	0	upwind
**(VO)**	222	11	7	0	downwind
	263	7	6	1	upwind
	263	24	23	1	downwind
	280	14	10	4	upwind
	280	15	14	1	downwind
	288	12	12	0	upwind
	288	24	19	5	downwind
	293	22	22	0	upwind
	293	17	14	3	downwind
	295	10	9	1	upwind
	295	16	12	4	downwind
	298	3	3	0	upwind
	298	10	9	1	downwind
	316	6	6	0	upwind
	316	19	15	4	downwind
	353	20	19	1	upwind
	353	15	14	1	downwind
	363	11	4	0	upwind
	363	4	8	3	downwind
	369	13	8	5	upwind
	369	14	8	6	downwind
	370	9	8	1	upwind
	370	6	6	0	downwind
	373	10	5	5	downwind

Plant material sampled from two orchards

**Fig 2:**
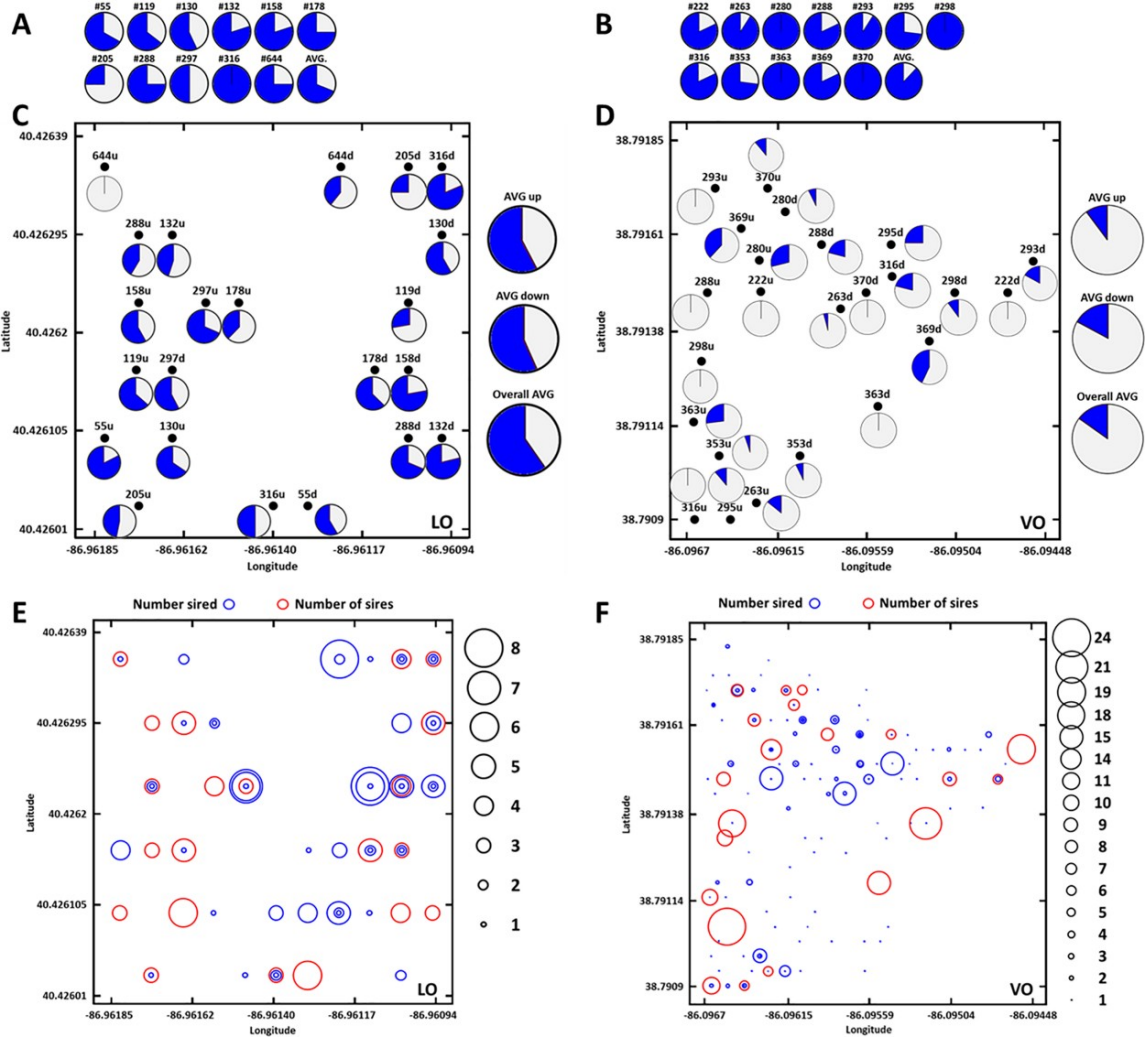
**(A, B)** For each clone pair (upwind, downwind), percent pollination from same male (grey), from different male (blue). **(C, D)** Location of maternal trees in both orchards (upwind, u; downwind, d) and proportion of pollen received from ‘inside’ the orchard (open) versus ‘outside’ (blue). **(E, F)** Number of sires (red circle) or number sired (blue circle). Number of sires accepted as pollen donors and number of times siring an offspring shown by increased circle sizes (1 = accepted from one male or sired one offspring, 24 = accepted from 24 males or sired 24 offspring. Red and blue together indicate a clone produced seeds and served as a pollinizer (sire) for another tree. LO = Lugar orchard and VO = Vallonia orchard.

* maternal parent clones tested which were validated with SSR markers; N = all progeny tested; number of progeny from within-orchard or wild pollination as determined by SSR genotype; upwind or downwind position in the orchards based on prevailing wind direction (see **Fig 2**).

#### Seedling production

Bareroot seedlings were tagged and lifted in the spring of 2013 and planted in a randomized incomplete block design at 2.2 m x 2.2 m spacing with 4-tree row plots, and each family had from 8 to 24 progeny per site. Seedlings were grown in two sites in Rush and Union Counties, IN, USA (**Table 1**).

### Microsatellite analysis, DNA extraction, and PCR

Genomic DNA was extracted using a CTAB protocol [22] from dormant twigs from all 48 grafted trees from LO and 158 grafted trees from VO during the 2013–2014 winter. DNA was extracted from young leaves from 23 families in the progeny tests (n = 884) in the summer of 2014. Trees were genotyped with 12 microsatellite (SSR) markers (WGA6, WGA24, WGA27, WGA32, WGA69, WGA72, WGA79, WGA86, WGA89, WGA90, WGA97, AAG01) [23] chosen for performance and ease of scoring [22, 24]. Parental trees were genotyped independently two times to confirm accuracy and reproducibility. All seedlings from each family were evaluated together in 96-well plates with their maternal clone to aid SSR scoring. Amplified fragment sizes were determined using an ABI-PRISM 310 genetic analyzer (Applied Biosystems, USA). Alleles were visually confirmed using Genemapperv3.7 (Applied Biosystems, USA). Polymerase chain reactions (PCR) were performed following procedures described by [25].

#### Genetic diversity and inbreeding index

Genetic diversity indices included the average number of alleles per locus (*A*), observed and expected heterozygosity (*Ho*, *He*), and polymorphic information content (*PIC*). Parentage and paternity analyses were performed using CERVUS v3.0 software [26]. The effective number of pollen donors within each orchard, paternity correlation, effective variance size, and effective neighborhood pollination area was calculated according to [21] where number of pollen donors, number of progeny, and proportion of seeds pollinated were the main parameters. We measured the co-ancestry coefficient within families using estimated paternity correlations and inbreeding coefficients [27]. The fixation index (*F*is) was estimated for orchard parents (*F*p) and seedling progeny (*F*s) using F = 1 - (*Ho*/*He*) [27]. All progeny were included in diversity estimates, irrespective of paternity, i.e., wild pollinated progeny were considered along with orchard pollinated progeny.

#### Paternity analysis

Paternity analyses were based on maximum-likelihood paternity assignments. The genotypes of all ramets in both orchards were determined. Each tree from which seeds were harvested thus had a known genotype for the female parent, and the genotype of the male (clone) could be determined and compared to the genotypes of orchard clones using CERVUS v3.0 software. Progeny that could not be matched to any male parent in the seed orchard were identified as wild. CERVUS v3.0 confirmed progeny pollen donors with analyses using all orchard trees as parents following methods described by [26]. Critical value simulations of Δ, the differences in likelihood ratios between the two males most likely to sire an offspring, for each confidence level in the paternity or maternity analyses (most likely parent), were conducted using CERVUS v3.0. Simulations used: 50,000 repetitions, 0.01 for proportion of loci mistyped, 95% relaxed confidence levels, and 206 male parent candidates for each progeny. Cervus v3.0 was also used to calculate exclusion probabilities.

There were 8 single unique genotypes (solitary clones and rootstocks) in VO that uniquely identified the male parent. Based on their orchard position, we were able to determine the angle and distance the pollen travelled.

Whenever multiple ramets of a clone were identified as a pollinator, we assumed the one nearest the female tree was the father. The position of these nearest males was then used to indicate pollen dispersal distance and to determine whether successful mating was a function of distance. Frequency distributions were applied to all families to examine the number of pollen parents in a family, and the relative proportion pollinated by each male.

Rose diagrams were created and analyzed using the **R** circular package [28] to illustrate distance and direction of pollen flow in each orchard. Moving outward on the radial scale, the direction and frequency of paternity can be visualized. The radial length of each spoke around the circle represents the percentage of time pollen blew from that direction. Parentage specifically shows the proportion of paternal relationships coming from a specific direction. Spokes are divided by color into distance and angles that pollen traveled. Rose diagrams were also produced with wind data from the flowering period in 2011 for comparison with pollen flow diagrams. Specifically, we used average daily wind from weather stations closest to each site (Bloomington, Monroe County Airport, 86 km from VO; Lafayette Purdue University Airport, 3 km from LO). Wind data were downloaded from the National Centers for Environmental Information. The proportion of pollen recipients and pollen donors, along with the total number of potential pollinizers, was visualized with MATLAB software [29].

#### Phenology

Phenology data was collected 2014–2016 in LO only based on methods described in [30]. First bud break was listed as the day when 50% of terminal buds opened. Data were recorded every 2 to 3 days and continued until the end of the bloom period. Male and female flowers were scored based upon first and last day of pollen shed or pistillate flower receptivity. Female and male flowers were defined as early, intermediate, or late. A clone was designated as early if its first flowers began to be receptive (females) or shed pollen (males) within the first week of flowering. Trees flowering within the week after early flowering clones were described as intermediate, and clones that flowered two weeks after the start of flowering or later were defined as late.

#### Statistics analysis

ANOVAs were analyzed using SAS/STAT software, Version 9.1 of the SAS System for Windows (SAS Institute Inc., Cary, NC, USA).

## Results

### Genetic diversity and inbreeding parameters

A total of 1,090 individuals from both orchards were genotyped for paternity analyses. Progeny with low quality amplification (n = 183) were removed. Two loci, WGA79 and AAG01, were removed due to non-specificity. All microsatellite loci analyzed were highly polymorphic (**Table 2**). SSR loci ranged from 5–20 alleles. Orchard clones had from 9 to 12 alleles per locus in LO and VO respectively. Progeny from both orchards averaged 13.4 alleles per locus. Average observed heterozygosity (*H*o) among orchard clones was 0.91 in LO and 0.79 in VO. Average expected heterozygosity (*H*e) was 0.81 in LO and 0.84 in VO; values for progeny pools from each orchard were similar (**Table 2**). Average fixation indices (*F*_*is*_) for LO parents were not significantly different than zero (**Table 2**). The highest inbreeding *F*_*p*_ was observed among parents in VO orchard (*F*_*p*_ = 0.141). There was also significant inbreeding among the progeny from LO (0.131) and VO (0.104) when wild pollinated progeny were eliminated. Indirect genetic similarity coefficients (Coancestry coefficient, *θ*_*xy*_) was 0.171 and 0.164 for LO and VO families, respectively (**Table 2**).

**Table 2:** Diversity, inbreeding and mating parameters estimates of seed orchard clones and progeny based on ten SSR loci.

Site	Source	N	K	*H*o	*H*e	*PIC*	*Fis*	*Nep*	*rp*	*θxy*	*NeV*
LO	**Clone**	15	9.1	0.91	0.81	0.76	0.080	-	-	-	-
VO	**Clone**	43	12.2	0.79	0.84	0.81	**0.141**	-	-	-	-
LO	**KM**	164	11.5	0.86	0.75	0.72	**0.131**	-	-	-	-
VO	**KM**	263	12.1	0.84	0.76	0.74	**0.104**	-	-	-	-
LO	**UM**	216	12.1	0.84	0.77	0.74	0.082	-	-	-	-
VO	**UM**	46	11.7	0.77	0.812	0.78	0.057	-	-	-	-
LO	**+**	380	13.4	0.85	0.77	0.75	0.097	3.96	0.25	0.171	2.91
VO	**+**	309	13.4	0.81	0.80	0.78	**0.100**	8.48	0.11	0.164	3.04

He, expected heterozygosity; Ho, observed heterozygosity; k, number of alleles detected per locus; PIC, polymorphism information content

KM, progeny with known male; UM, Progeny with unknown male (sired by wild trees). **+**, all seedling sired by known and unknown males; F(is), fixation index for parent and progeny; N_ep_, The effective number of pollen donors mating with each tree, n_ep_,was the number of pollen donors detected from paternity results, where n_p_ was the number of progeny analyzed, P_i_ was the proportion of progeny sired by male i (Bittencourt et al., 2007); rp, paternity correlation among offspring of the average seed tree (rp: 1/Nep; Bittencourt et al., 2007); θxy, coancestry coefficient = θxy, 0.125 (1 + Fp) (1 + rp); NeV, variance effective size = NeV, 0.5/θxy; *p < 0.05 (Bittencourt et al., 2007).

### Parent progeny relationships / isolation

All parental genotypes matched their indicated reference clones except for one tree in LO (a rootstock) and eight trees in VO (single surviving grafts and rootstocks). Progeny were genotyped and matched their maternal clones at 99.9% (LO) and 98.0% (VO). Paternity exclusion probability based on microsatellite allele frequencies was 0.98 (LO) and 0.97 (VO). We found no evidence of self-pollination in progeny from either orchard. Overall, 434 (62%) of 699 progeny were assigned pollen parents at a confidence level of 95%. Not all clones sired offspring. Paternity analyses determined that 43% of clones at LO and 85% of clones at VO were male parents. Clones 132, 293, 348, and 364 each sired a single seedling in VO. At LO, clone 119 pollinated 32 progeny or 20% of all progeny of eight different maternal clones. At VO, clone 295 pollinated 24 offspring of 11 clones (10% of all progeny). Four clones pollinated 50% of LO progeny, whereas 18 clones pollinated 50% of VO progeny. Differences in total pollen donor numbers between orchards were highly significant (*p* < 0.001). The average effective number of pollen parents per ramet, per clone, averaged 3.96 (ranging from 1.25–6.25) in LO, and averaged 8.48 (ranging from 1.8–13.44) pollen parents in VO. Both ramets of each maternal clone were pollinated by the same male clone 31% of the time at LO and 12% at VO, a difference that was highly significant (*p* < 0.0001) (**Fig 2A and 2B**).

Pollen parents were confirmed for 162 of 380 progeny (42.7%) from the non-isolated LO. Pollen parents were confirmed for 272 of 318 progeny (85.4%) from the isolated VO. Isolation proved highly significant (*p* < 0.0001) for decreasing wild pollination and increasing within orchard pollination.

### Within-orchard pollen flow based on prevailing wind direction and distance

**Fig 3:**
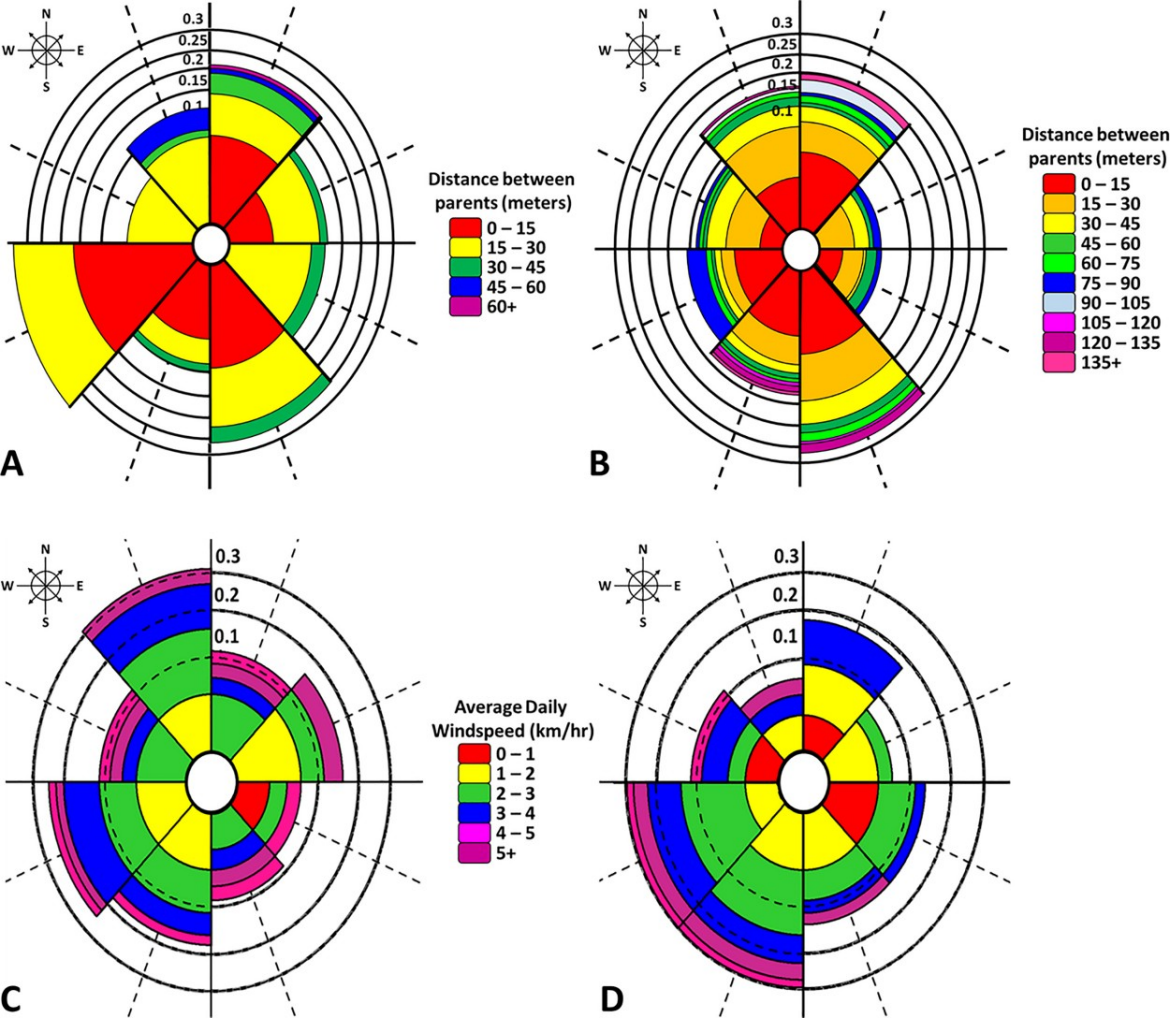
Rose diagrams showing frequency, distance, and direction of pollen that resulted in production of an offspring. The observed distribution of successful mating was divided into eight sectors (**A**) Lugar farm orchard (LO) and **(B)** Vallonia orchard (VO) obtained from pooled data and all verified mating events. Direction and speed of wind (**C**) West Lafayette, Indiana and (**D**) Bloomington, Indiana, the closest weather stations to LO and VO. Scale shows: 0.1 = 10%, 0.15 = 15%, 0.2 = 20%, 0.25 = 25% and 0.3 = 30%.

**Fig 4:**
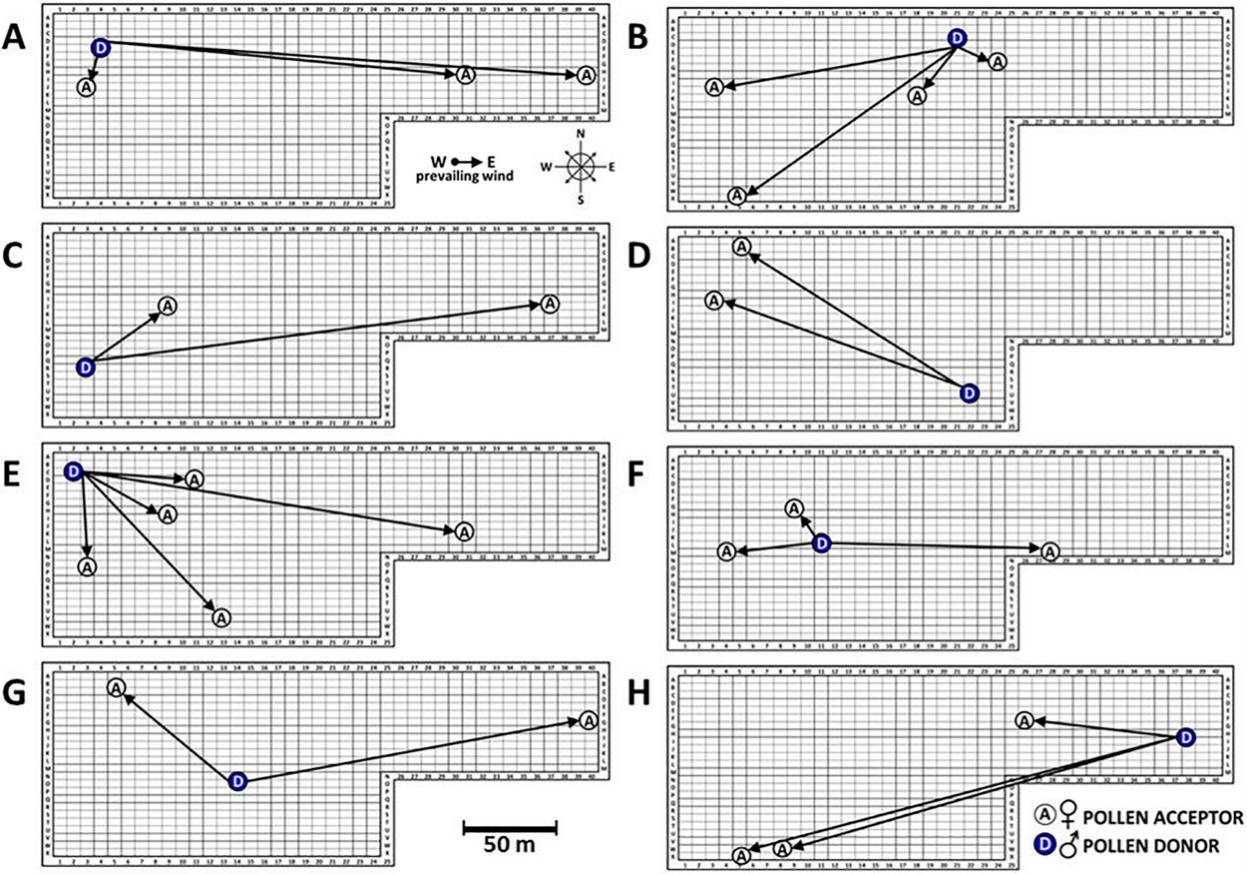
Selected single tree genotypes illustrate distance and angle of pollen flow in VO based on paternity analysis. **(A)** Clone 297: pollen donor for clone 288, 294, and 293. **(B)** Clone 139: pollen donor for 295, 263, 295, and 288. **(C)** Clone 192: pollen donor for clone 222 (upwind and downwind). **(D)** Clone 332: pollen donor for 293 and 288. **(E**) Clone 289: pollen donor for 370, 298, 280, 353, and 298. **(F)** Clone 363: pollen donor for clone 280, 373, and 369. **(G)** Unknown genotype inside orchard pollinated 293 (upwind and downwind) **(H)** rootstock tree that pollinated ramets of clones 283, 263, and 295.

Each female was represented by two ramets with one located in an upwind and the other in a downwind position. Fifty percent of upwind clones and 52.5% of clones downwind received pollen from wild trees in LO, a difference that was not significant (*p* < 0.12). (**Fig 2C and 2D**). There were too few wild pollinated progeny at VO to statistically estimate but the trend was similar. Rose diagrams and data from pollen donor genotypes present in the orchard as single genotypes showed no effect of prevailing wind direction on pollen parentage in either orchard (**Figs 3 and 4**).

Pollen travelled within both orchards, on average, 50 m. Roughly 83% of pollen traveled up to or less than 50 m and only 5% of pollen traveled 90 m or more. Within the orchards, maternal and paternal trees ranged from 6 m to 166 m apart. (**Fig 3**).

### Pollen flow based on phenology data

**Fig 5:**
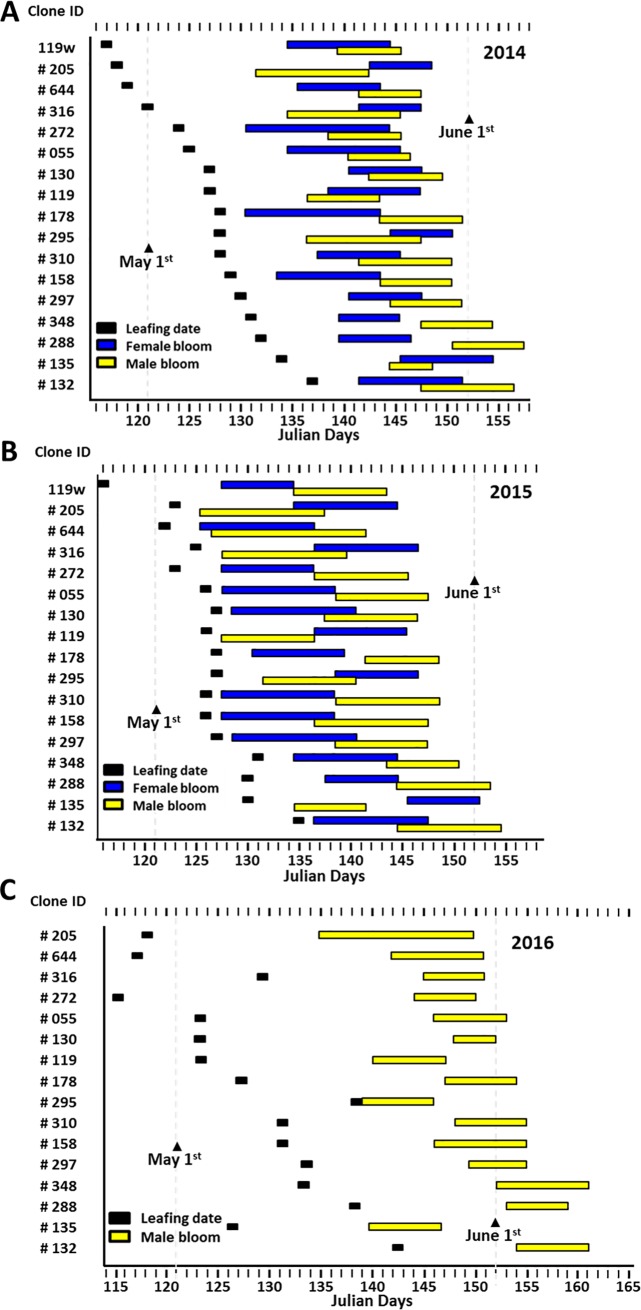
Leafing dates and bloom phenology of *J*. *nigra* in LO (**A**) 2014, (**B**) 2015 and (**C**) 2016. 119w (in **A**, **B**) is the rootstock tree.

**Fig 6:**
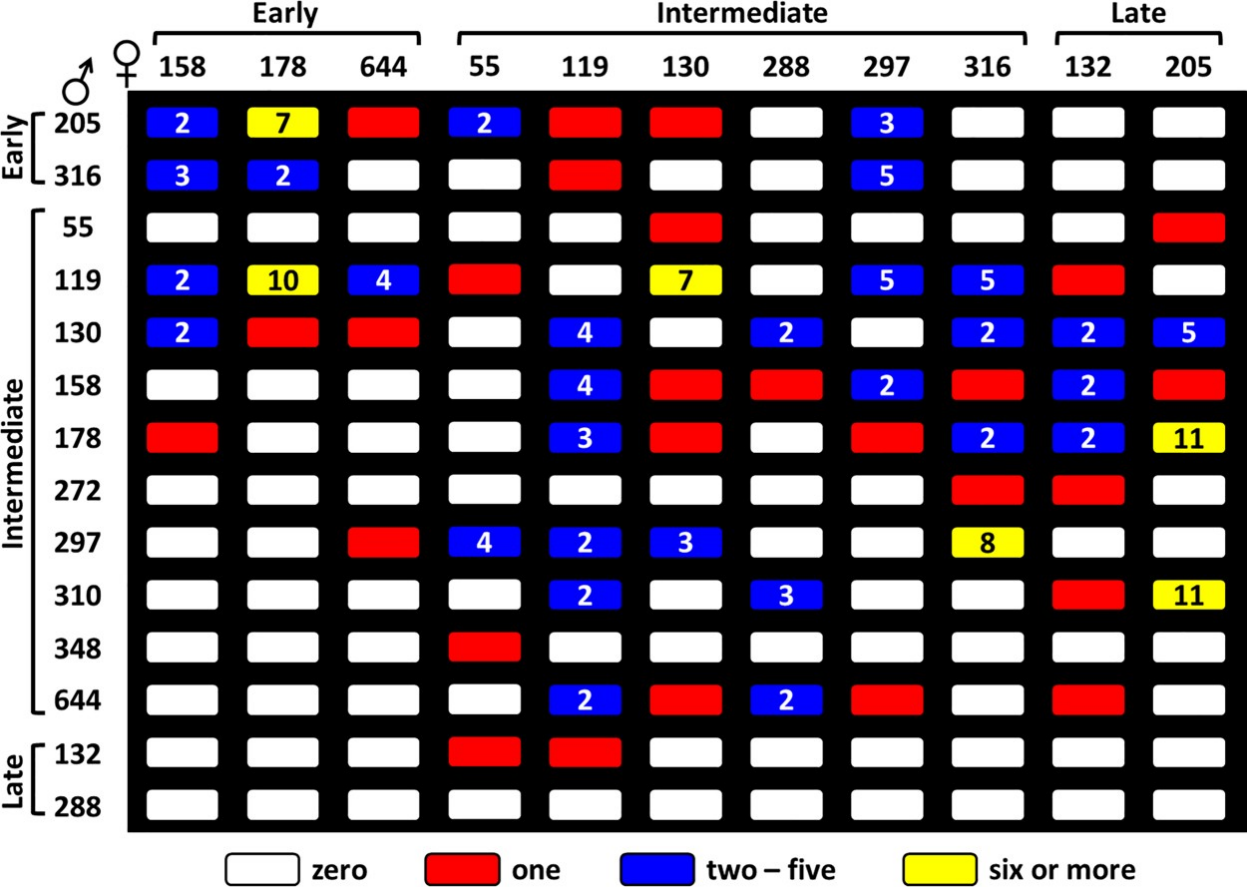
Cross pollination based on timing of female and male flowering in Lo orchard.

Phenology data were collected in 2014, 2015 and 2016 from LO only (**Fig 5**). Female flowers were observed on Julian day 130 in 2014 and started three days earlier in 2015. In 2016, insects destroyed terminal buds where the pistillate flowers are born and no female flowers were observed that year. Male flowers shed pollen from Julian day 132 to 158 in 2014 and from Julian day 127 to 155 in 2015, and from Julian day 132 to 162 in 2016. Average duration of flowering per clone was 8 days (female) and 7 days (male). We observed a significant positive correlation between average leaf out date and male flowering time (Spearman’s r = 0.71, *p* = 0.0005) in 2014 and 2015, but not between leaf out and female flowering time (0.50, p = 0.099) in 2014 and 2015 (**Fig 5**). Although time of flowering changed year to year, most ramets of all clones were consistently early, intermediate or late blooming across all years (**Fig 6**).

Based on the phenological groups listed in **Fig 6**, for LO, early blooming maternal clones (n = 3), received 1% of pollen from early blooming male clones; 76% from intermediate, and 23% from late blooming paternal parents. Intermediate maternal clones (n = 6) mated 11.5% with early, 86% with intermediate, and 2.5% with late blooming paternal clones. Late maternal clones (n = 2) produced 100% of their offspring by mating with only intermediate male paternal trees (**Fig 6**). There was a significant association between percent of offspring from a particular male clone and maternal phenology group (*χ2* = 22.43, *p = 0*.*0001*). No late blooming paternal trees pollinated any late blooming maternal trees. Our results revealed early and late maternal clones received little or no pollen from early or late paternal clones.

## Discussion

### Genetic diversity and fixation indices

Despite the differences in number of parents between orchards (LO, n = 15; VO, n = 37), both exhibited relatively high levels of genetic richness based on percent heterozygosity and allele numbers per locus (**Table 2**). The high level of genetic richness and heterozygosity among the orchard parents was reflected in high exclusion probabilities for paternity analysis, values similar to those [31] reported for wild black walnut. Black walnut is a predominantly outcrossing species, but we found no evidence of selfing from either orchard. Studies of other *Juglans* species, including *J*. *mandshurica* and *J*. *ailantifolia*, reported selfing rates of 3.8% and 15% [32, 33] based on fertilized seed samples. Because we sampled seedling progeny in their third year in the field, it is likely that any self-pollinated seeds failed to germinate, were too small and culled out of the nursery, or died within two years after planting before we sampled. [31] Found very few self-pollinated black walnut progeny utilizing a similar approach to the present study.

There was a positive and significant fixation index value for VO orchard parents (**Table 2**), which indicated some inbreeding among that population. While not statistically significant, a positive fixation index was also found for VO progeny. As VO orchard parents were forward-selections derived from open pollinated progeny, it is likely that some of these forward selections shared male parents. In contrast, fixation indices from the backward selected LO parents and their progeny were insignificant as LO parents were all unique and unrelated genotypes (**Table 2**). It was reported that in wild populations of *J*. *mandshurica* the coefficient of inbreeding was higher for progeny than for parents but the difference, while significant, was minimal [32, 33].

### Pollen contamination in isolated verses non-isolated orchards

In the isolated VO, wild trees pollinated 14% of the progeny while at LO, 57% of the progeny were pollinated by wild walnuts. The wild walnut trees near LO were older and much taller at 28 m compared to 13 m respectively, and likely produced considerably more pollen that dispersed over a greater distance. We found significantly less wild pollination in VO than was reported in a study of a wind-pollinated population of Norway spruce (*Picea abies*) isolated by 1 km [34] but higher levels than a study of limber pine (*Pinus flexilis*) isolated by more than 1 km. Outside-pollen was estimated to be 6.5% in a limber pine orchard isolated by 5 km [35] and 4.3% in a Scots pine (*P*. *sylvestris*) orchard isolated by 30 km [36]. In contrast, orchards planted near conspecifics showed 31% outside-orchard pollination for Japanese red pine (*P*. *densiflora*) isolated by just over 100 m [18]. Results from bur oak (*Q*. *macrocarpa*) forest stand isolated by 200 m indicated pollen contamination approached 57% [18], a level of contamination similar to that found in LO. The amount of pollen contamination in Japanese walnut (*J*. *ailantifolia*) was 47.9% [33]. Pollen contamination in a small black spruce (*P*. *mariana*) seedling seed orchard for three consecutive years was 32%, 83% and 58% percent respectively [37] showing how pollen dynamics can vary from year to year.

Based on prevailing wind direction, we hypothesized that pollen from wild trees would be a larger proportion of the pollen cloud available to the “upwind” trees, resulting in a higher percentage of seedlings with wild male parents than “downwind” trees would have. But only a 2.5% difference occurred at LO. Neither proximity to wild trees outside the orchard, nor position relative to other ramets of the same clone in the orchard, had a discernible effect on levels of contamination in LO (*p* < 0.12). In contrast, studies of mixed oak (*Quercus spp*.) stands revealed 66% of offspring were pollinated from males located outside the stand, with significant correlation between position and level of gene flow [20], showing that in some cases, pollen flow significantly varied due to a trees position in the stand.

### Pollen flow distance and direction

In general, we found no clear pattern of pollen movement at either orchard due to orchard position and overall, 83% of pollen traveled 50 m or less, ranging from 6 to 166 m (**Figs 3 and 4**). The average distance between mates in our study was higher than *J*. *mandshurica* (15 m; [32]) and knobcone pine (*P*. *attenuata*) (5.43 m; [21]), but comparable to English oak (*Q*. *robur*) (22 m–58 m; [20]), sessile oak (*Q*. *petraea*) (18 m–65 m; [20]), northern red Oak (*Q*. *rubra*) (60 m; [10]), and Japanese red pine (*P*. *densiflora*) (68 m; [17]). Our 50 m average distance was significantly lower than several other pines and one hardwood: limber pine (*P*. *flexilis*) (140 m; [35]), European ash (*Fraxinus excelsior*) (328 m; [38]) and Parana pine (*Araucaria angustifolia*) (2200 m; [39]). In *Juglans* (*J*. *regia*, *J*. *nigra*, and *J*. *mandshurica*), pollen was reported to travel from 140 m to 1440 m [5, 31, 32]. Therefore, compared with other wind-pollinated species, black walnut can disperse pollen over relatively long distances within the orchard, however, short-distance pollen movement predominates, much like [5] reported for Persian walnut orchards.

Pollen dispersal dynamics have rarely been studied in forest trees given the complexity of such environments. Our results agree with other studies that found prevailing wind direction failed to influence pollen movement [18, 21, 40]. In general, and based this study of two orchards, clone placement based on prevailing wind patterns in a seed orchard is not critical. Seed orchard design for black walnut can focus instead on placement of parents alone without consideration of prevailing wind direction.

The likelihood of pollination by the same male parent for two ramets of the same maternal clone was 31% at LO and 12% at VO (**Fig 2A and 2B**). LO had only 15 clones while VO had 37 clones. With fewer parents shedding pollen at any given time at LO, seed parents were pollinated by the same clone more than twice as often as seed parents in VO. These data support previous observations that pollen donors closest to a receptive female are likely to be the most frequent pollinator of that female's offspring. Nevertheless, we observed that parents of trees in VO were rarely immediate neighbors (**Fig 4**). Density of pollen dispersed from each tree declines rapidly as distance increases, and can be modeled using a negative exponential distribution [33, 41] because pollen is diluted in air as it travels away from its source [18]. [42] Reported that the nearest 25 males around a mother tree sired 60% of progeny, and [21] reported 41% of progeny were the result of pollination between neighboring trees.

### Number of pollen parents

Clone 119 pollinated 8 of 11 clones at LO and clone 295 pollinated 10 of 12 clones at VO. Half of all progeny were pollinated by 4 males in LO and 18 males in VO. LO families were pollinated by 1 to 8 males and at VO, with 43 genotypes, families were pollinated by 1–15 males (**Fig 2E and 2F**). In a *Quercus robur* clonal seed orchard, [43] reported that the number of pollinations by clones was highly related to the number of ramets of that clone in the orchard. Unequal male reproductive success in open-pollinated seed orchards has been widely reported; e.g., red oak (*Q*. *rubra*, [10]), Norway spruce (*Picea abies*, [44]) and red spruce (*P*. *rubens*, [45]). [46] determined that 80% of progeny from a seed orchard containing 12 silver fir (*Abies alba*) clones were sired by just four clones. Similarly, two earlier studies of Nordmann fir (*A*. *inordmanniana*) revealed that 3 of 13 clones sired 75% of progeny [47] and 5 of 23 clones sired 60% of progeny test offspring in a separate test [48]. Three of six *Eucalyptus urophylla* clones in a production seed orchard sired 148 of 149 sampled offspring in one progeny test [49], and 199 of 349 (57%) potential male trees sired 440 offspring at variable rates in *Eucalyptus* (*E*. *grandis*) [50].

Unequal male reproductive success could lead to decreased genetic diversity and inbreeding which can lower survival and fecundity. Use of a mix of different pollen parents, or a “polymix,” [14, 51] to pollinate isolated seed parents has been used with good effect to control these limitations. These authors noted that addition of paternity testing, as in our present study, is an effective way to monitor pollination dynamics and, depending on the choice of male parents, can further mitigate inbreeding.

### Phenology analysis

Phenological markers such as the timing of bud-break, female and male bloom times and bloom duration vary yearly depending on local temperatures and winter chilling. We found that the period of pollen shed lasted 25 to 30 days over three consecutive years in LO. In Missouri, bud break among 21 black walnut cultivars over four years varied from 26 to 47 days [52]. Among-year variation in bloom dynamics influences the degree of random mating (panmixia) in a seed orchard, and SSR markers can provide a good monitoring tool for open pollination breeding. [10] Studied bloom phenology in northern red oak seed orchards for two years and observed that paternity dynamics varied only slightly from year to year.

Bloom phenology (early, intermediate, or late), in general, was a poor predictor of mating pairs in LO. Paternal clones that shed pollen during the intermediate bloom period pollinated more seedlings compared to early and late blooming clones. Early and late trees, which rarely mated with each other, were both receptive to intermediate pollen and highlighted the importance of having surfeit receptive parents throughout the flowering period. We also noted early and late flowering clones were more receptive to wild pollen than intermediate clones. Knowing such dynamics would help improve the timing of supplemental pollen application, for example, by focusing on the early and late bloom period rather than the intermediate period.

### Implications for black walnut breeding

This is the first large-scale examination of molecular genotyping used to identify open-pollinated black walnut timber family paternity. Open-pollinated half-sib breeding of black walnut can be improved utilizing DNA SSR markers to identify pollen parents. We found that isolation from other black walnut led to 85% within-orchard pollination in contrast to 42% within-orchard pollination from our non-isolated orchard. Now that we have genotyped LO and VO in their entirety, we can determine the male parent of selections in other progeny tests derived from these two orchards. New black walnut timber seed orchards should consider having at least 40 parents and be isolated from other walnuts to create a “polymix-like” open pollinated population.

While VO had many more parents than LO, (37 versus 15 clones), VO parents showed a slight yet significant level of inbreeding while LO parents did not. The difference likely results from the fact that VO parents were forward selections from progeny harvested from a single grafted clone bank. In contrast, LO parents were primarily first generation backward selections. As [51] point out, some inbreeding can help promote accumulation of desired alleles. In fact, [53], compared the VO population to the original Purdue clone bank and other natural black walnut seed populations and found significant timber quality differences among all populations after 10 years in the field with the VO population ranking highest for timber quality followed by the Purdue clone bank. These authors also found no statistically significant differences in height or diameter growth at 10 years but the VO population ranked first in seedling size followed by the Purdue population and five other wild populations.

Since most pollination occurred within 50 m, future orchards could be planted with single ramets of clones completely randomized to ensure all parents can cross pollinate. Given the practical difficulties of artificially pollinating black walnut in the field, and the expense and resources required to grow large grafted seed parents in a pollen-free greenhouse, “breeding without breeding” using DNA markers, is an excellent and effective method for advancing black walnut timber breeding.

## Supporting information

S1 Table Microsatellite data used for paternity analysis. (XLSX)
